# Antibodies for immunolabeling by light and electron microscopy: not for the faint hearted

**DOI:** 10.1007/s00418-014-1263-5

**Published:** 2014-08-24

**Authors:** Gareth Griffiths, John Milton Lucocq

**Affiliations:** 1 Institute of Biological Sciences, University of Oslo, Blindern, 0316 Oslo, Norway; 2 Schools of Medicine and Biology, University of St Andrews, North Haugh, Fife, KY16 9TF Scotland, UK

**Keywords:** Antibodies/Specificity, Immunocytochemistry, Stereology/Quantitation, Commercial antibodies, Light microscopy/EM

## Abstract

Reliable antibodies represent crucial tools in the arsenal of the cell biologist and using them to localize antigens for immunocytochemistry is one of their most important applications. However, antibody–antigen interactions are much more complex and unpredictable than suggested by the old ‘lock and key’ analogy, and the goal of trying to prove that an antibody is specific is far more difficult than is generally appreciated. Here, we discuss the problems associated with the very complicated issue of trying to establish that an antibody (and the results obtained with it) is specific for the immunolabeling approaches used in light or electron microscopy. We discuss the increasing awareness that significant numbers of commercial antibodies are often not up to the quality required. We provide guidelines for choosing and testing antibodies in immuno-EM. Finally, we describe how quantitative EM methods can be used to identify reproducible patterns of antibody labeling and also extract specific labeling distributions.

## Introduction

The use of antibodies to identify the localization of antigens in cells and tissues has long been one of the most powerful and popular tools in cell biology and related disciplines. When one knows for sure in which cells, and then in which organelles an antigen resides, one acquires crucial information about the antigen and its potential functions. The theory is ‘clean’: one makes an antibody against, say a purified protein and the antibody is supposed to bind to the antigen like a key fits a lock. It should thus be technically straightforward to add the antibody to, say a section of cells or tissues and identify unequivocally where the antibody binds; which should be the site(s) where the antigen is localized. As discussed in more detail below, since there are now dozens of companies selling thousands of antibodies against a huge number of proteins (and other antigens such as lipids and nucleic acids) at first glance the situation ‘smells like a rose’. A beginner in the field can be easily fooled by this appealing scenario, unaware that a more realistic cliché might be—a ‘hornet’s’ nest’!

It is a long and unpredictable route from an antigen that needs to be prepared for injection into an animal (most commonly a rabbit or a mouse) to the serum or purified antibody that is ready to be used for immunocytochemistry (ICC); and therefore, antigen X ‘in’ does not necessarily mean anti-X ‘out’. A complex, nonlinear path of cell interactions can be traced from the antigen-presenting cells that first take up the immunogen, via T helper cells through to B lymphocytes. The B cells can be sorted into individual clones, each derived from a single cell that secretes identical antibodies. When the antibody is made by a single clone it is referred to as a monoclonal antibody. When a mixture of clones is used, as is the case with virtually all antisera, the antibody is a polyclonal antibody. Both types of antibodies are widely used for ICC. A beginner is well advised to read the excellent monographs on how to make and characterize antibodies by Harlow et al. (1988) and Frank (2002) that are especially relevant for use in immunocytochemistry. For general reviews on immunocytochemistry procedures and theoretical background see Ramos-Vara (2005), Dalcik and Dalcik (2012) and Griffiths et al. (1993).

It must be appreciated from the outset that only a minority of antibodies are useful for immunocytochemistry. Looking at the complexity of the interactions that are necessary for making an antibody, it may not be so surprising that the success rate is far from perfect. A glimpse at the details of how antibodies actually bind protein antigens makes it evident that the situation is even more complex. According to Frank (2002) an epitope in a protein may encompass about 15 amino acids but only about five of these contribute to the binding energy. On the other hand, the part of the antibody that binds the epitope is the paratope, a part of the antibody variable region, which comprises about 50 amino acids. Similarly, each paratope has about 15 amino acids of which about five contribute to the binding energy for the epitope. Frank stated that ‘Paratopes and epitopes define complementary regions of shape and charge rather than particular amino acid compositions’; and ‘A single paratope can bind unrelated epitopes and a single epitope can bind to unrelated paratopes’ (Frank 2002). Thus, it is possible that a single paratope can bind two unrelated peptides (mimitopes), or a particular epitope can be recognized by two different paratopes with no sequence similarity.

A serious consequence of these facts is that an antibody against a defined antigen, e.g., a whole purified protein or a peptide, could bind to structurally related antigens that have a completely or partially different amino sequence (molecular mimicry). This means that, predicting an antibody has high affinity for the immunizing antigen is extremely difficult if not impossible. Such molecular mimicry has been convincingly demonstrated for antibodies made against well-defined, small molecule chemicals such as 2,4-di-nitrophenol (DNP), a favorite of immunologists. Varga et al. (1991) tested a mouse anti DNP-IgE antibody (SPE7) against a library of over 2,000 compounds and identified a number of unrelated chemicals which were able to compete with DNP for binding to SPE7. In a more detailed study of the same antibody by James and Tawfik (2003), it was confirmed that the antibody had a high affinity to DNP, but not to closely related chemicals. However, a number of totally different chemicals were also able to bind with high affinity and they selected a few for X-ray crystallographic studies of the antigen–antibody complexes. Crucially, this revealed that each chemical bound in a specific manner with different stereochemistry by hydrogen bonding to different residues located within the antibody binding site. Thus, even with these small, well-defined, molecules the ligands that would bind specifically could not be predicted a priori.

There are also multiple examples of similar cross-reactivity of antibodies made against a particular protein that cross-react, seemingly specifically, to both related and unrelated antigens. For example, Frank (2002) cites Lescar et al. (1995) who used X-ray crystallography to study the physical contact site between guinea fowl lysozyme and two different antibodies. While both these antibodies engaged with the same 12 amino acids of the antigen, they contained two different paratopes—with no identical amino acids in the region that binds the antigen. Crucially, the two antibodies also had different patterns of cross-reactivity.

If the situation is so unpredictable for antibodies binding to simple antigens, it can be appreciated that we are approaching ‘no man’s land’ when it comes to the use of antibodies on sections or whole mounts that have been subjected to the conditions needed for immunocytochemistry (aldehyde cross-linking, embedding, antigen retrieval, etc.). The use of specificity tests discussed below has nevertheless identified clear examples of antibodies that bind in a way that can be described as ‘specific’, by many criteria to antigens totally unrelated to the initial immunizing antigens. For example, Holmseth et al. (2005, 2006, 2012) made dozens of different antibodies against transporters of the excitatory neurotransmitters EEAT2 and 3 (these studies revealed a rich treasure of problems related to the topic of this review, using all the best controls). Relevant here is their example of anti-peptide antibodies raised against EEAT3, of which some cross-reacted with high affinity to an unrelated protein tubulin. Importantly, while these anti-tubulin antibodies could be removed effectively by affinity purification against tubulin, one antibody retained specific binding to both EEAT3 peptide and tubulin after this treatment. It should be noted that the adsorption method is only applicable to polyclonal antibodies; monoclonal antibodies are by definition identical.

A number of examples have been published illustrating how aldehyde fixation used for microscopy induces the presence of new epitopes in sections that are unrelated to the starting antigen. A striking example was described by Josephsen et al. (1999). These authors showed that a monoclonal antibody against vimentin showed a selective and strong cross-reaction to unrelated tooth enamel proteins, amelogenins. A series of experiments made the convincing case that the ‘new’ reactivity was induced by aldehyde cross-linking of the tissue; this notion was supported by experiments in which fixed and unfixed Western blots were compared before antibody labeling.

Another excellent example of the subtle issues of antibody specificity comes from the work of Watanabe et al. (1998). These authors addressed the localization of the NR2A glutamate receptor in the mouse hippocampus. When they initially compared aldehyde-fixed sections of brain tissues from wild type (WT) and mice knocked out (KO) for NR2A they saw a similar cytoplasmic labeling by immunofluorescence labeling of both tissues. However, when they tested the antibody after treating the sections with proteases (pepsin or proteinase K), they saw what was interpreted as a specific membrane-bound labeling in the WT that was missing in the KO sections. A similar example of apparent specificity of antibody that was revealed for immunofluorescence on sections of mouse cortex after pepsin treatment was described by Lorincz and Nusser (2008) for a potassium channel protein Kv1.2. These authors speculated that the protease treatment removes proteins that block access of the antibody to the specific antigen in the sections; this is an example of ‘antigen retrieval’, commonly used approach for, e.g., neurobiology and pathology (but not often in cell biology). The main take home message here is that success in providing convincing evidence that an antibody is specific for an antigen can be unpredictable.

In summary, rather that representing the classical view of one antibody binding to a protein like a key fits a lock, these, and many other examples we have not cited, lead us to the conclusion that the ‘key’ may fit many types of ‘locks’ and the lock can fit an array of different keys.

## The problems with primary antibodies

Our focus here is on the primary antibodies that are the main problem when assessing specificity; for a good, concise introduction to the problem see Saper (2009). In most protocols, the primary antibody is detected by a generic second antibody attached to a visualizing marker. For example, a rabbit primary antibody against X can be detected by a goat anti rabbit secondary antibody conjugated to a fluorochrome or a colloidal gold conjugate. Obviously, it is also important that the secondary antibodies are specific but, in practice, this is far less of a problem than the issue of primary antibody specificity. The key control is to add all the secondary, and tertiary reagents in the absence of the primary antibody.

The concern with secondary antibodies, especially when used for double labeling, is inter-species cross-reactivity. Reagents are available which are specifically adsorbed against immunoglobulins of other species. However, cross-reactivity, in particular with rabbit antibodies to infectious agents such as *Mycobacterium* spp. and *Rhodococcus* spp., is a serious issue and in our experience cannot be reduced, not even with pre-adsorption against these organisms or their homogenates (Urska Repnik and G. Griffiths, unpublished data). Some of these problems may be due to mycobacterial cell wall components in Freund’s adjuvant that used to be commonly used in preparing polyclonal antibodies. However, we have also seen cross-reactivity with monoclonal antibodies where this adjuvant was not used.

For any ICC approach for light microscopy or EM, the cells and tissues need to be subjected to a series of preparation protocols, including fixation, detergent permeabilization, embedding, sectioning before the antibody reaction steps. The examples above of significant effects of aldehyde fixation and antigen retrieval on immunolabeling is only a glimpse into a whole world of complexity in which all the preparation steps can positively or negatively influence the final result and the reliability of that result. A key issue here in dealing with ICC is providing evidence about specificity which is based as much as possible *on the actual conditions in which the antibody labeling is carried out*.

**Fig. 1:**
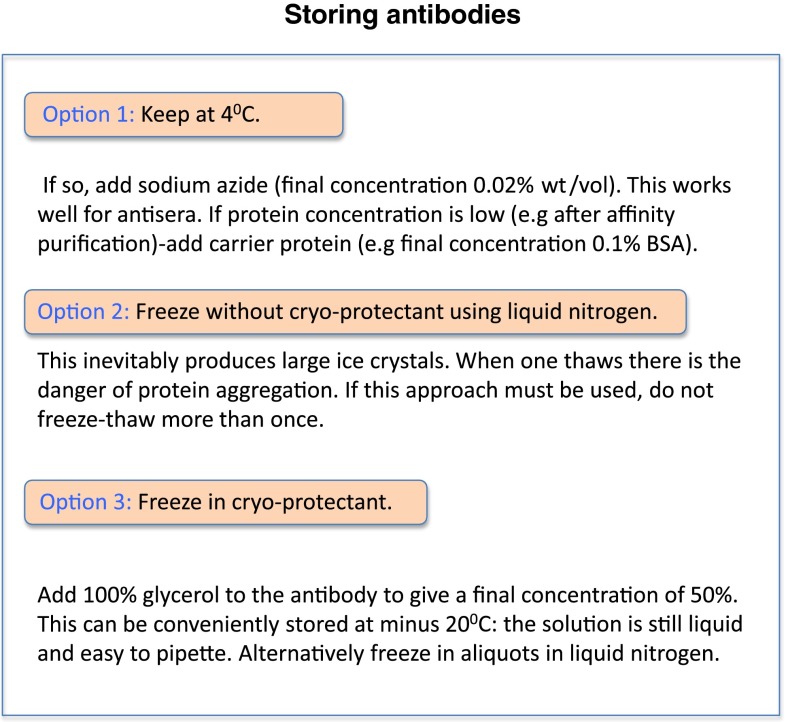
Tips on antibody storage

Another crucial issue that is often not taken seriously enough is that a solution of IgG in a test tube is not a simple chemical but a complex protein that can aggregate, be subjected to proteolysis, undergo denaturation or stick to the surface of the tube. Even under the best conditions, antibodies can lose activity with time, further complicating the issue of deciding when an antibody labeling is ‘real’. In one’s own lab one has some control over this issue but with commercial antibodies (see below) one is at the mercy of the company. Figure 1 gives some suggestions as to how antibodies should be stored.

Transporting antibodies in tiny aliquots is also a problem. An excellent system for storing and transporting antibodies, even in 1 μL aliquots has long been used by Heinz Schwarz (personal communication); this involves flame-sealing the antibody in glass micropipettes. These can be stored for many years at −20 °C.

The problem of providing (preferably unequivocal) evidence justifying the assumption that an antibody used for ICC is ‘specific’, is analogous to the problem that the police and the justice system have in identifying a potential murderer, and then in proving ‘beyond reasonable doubt’ that the accused is indeed guilty of the crime. The system needs to take into account all available evidence that supports the hypothesis that the accused is guilty, or not. Some evidence, such as a motive might be given less weight than, say solid DNA-based evidence found at the scene of the crime, but it is still the sum-total of all the evidence which the judge or the jury uses to make a decision.

So it is with antibodies. It is often very difficult, if not impossible to prove beyond all reasonable doubt that, *under the conditions used for the ICC reactions*, a particular antibody is specific for the true target antigen. It is therefore important to collect supporting or contradictory evidence from all possible sources. Some of this can be given more weight than others. We will summarize the main types of evidence that can be used.

## Criteria that can be used to assess specificity and the potential problems associated with them

1. The antibody against antigen X gives a reproducible and compartment-selective labeling pattern. This is a crucial starting point because without this, there is nothing to evaluate. The pattern may not be readily discernable by ‘eye’ and below we describe quantitative methods available for finding patterns in immunogold labeling.
  Problems: *The labeling may be associated with structures not containing the real antigen*.
2. This labeling pattern should be consistent with information about the antigen from other sources. For example, the presence of a signal sequence and membrane-spanning domain would indicate it should be found in membranes.
  Problems: *The biological information may be wrong or misleading*.
3. The labeling is lost in a knockout (KO) animal or cell reduced in a knock down situation (e.g., using si or sh RNA). As discussed in more detail below a consensus is emerging that this is the most informative control, when available.
  Problems: *Deletion of a gene may lead to up*-*regulation of related gene products that might cross*-*react with the antibody. There may already be cross*-*reacting antigens in the KO animal. There may be changes in cellular structures or compartments that confound the identification or quantification of structures or compartments. It is obviously important that the KO procedure does not lead to expression of non*-*functional proteins.* For subtle, predictable and unpredictable artifacts see Holmseth et al. (2006, 2012), Burry (2011), Lorincz and Nusser (2008). It should be noted that all antibodies, including monoclonals can show cross-reactivity to off-target antigens (Holmseth et al. 2012; Nigg et al. 1982).
4. If the protein can be expressed or over-expressed in a cell or organelle not expected to contain the antigen, there should be a corresponding increase in labeling in some structures (Saper 2009). This control is seen as comparable to the KO control.
  Problems: *Artificial expression and even more likely, over*-*expression may lead to mis*-*localization of the protein.* For example, a peripheral membrane protein may become displaced to the cytoplasm (Soderqvist et al. 1996). *Again there may be modifications to the very compartments that are used for the evaluation including those that really hold the antigen and those that don’t. Changes in these compartments can then produce changes in density or quantity of the labeling signal.*
5. If the protein can be expressed as a GFP construct the pattern of GFP expression should correlate with the labeling of an antibody against the same protein (Burry 2011). The use of an antibody against GFP can also be a powerful tool in this context. Other tags such as myc, FLAG or HA may be used instead of GFP. The use of cell lines stably expressing these tagged constructs at low levels can reduce artifacts of overexpression. The tags can also be expressed as an RNAi resistant tagged construct while at the same time allowing parallel knockdown of the endogenous background expression.
  Problems: *There is no guarantee that the GFP*-*linked protein will localize faithfully, especially when overexpressed.*
6. Independent methods for separating the antigen from other antigens, derived from the cells or tissues of interest, such as one-dimensional or two-dimensional Western blots (WB) or thin layer chromatography, should give an identification pattern on the separated antigen (e.g., the expected molecular weight band(s)) that is consistent with the known biology/biochemistry of the antigens.
  Problems: *There is the fundamental problem that WB recognizes SDS*-*denatured antigens whereas aldehyde*-*fixed proteins for immunocytochemistry are closer to being in the native state* (Griffiths et al. 1993). There are many examples of WB giving results that are mis-leading for interpretation of antibody specificity for immune-labeling (Holmseth et al. 2005, 2012). Almost all commercial antibodies give some kind of reactivity by WB but far fewer (are claimed to) label for ICC. A single band by WB may have dozens of different protein species when analyzed by mass-spectrometry (Thiede et al. 2013) or multiple spots on 2D gels. Additional problems associated with gel separation methods in general is that only those antigens that enter the gel and separate from other components are identifiable. For a detailed discussion on the use and pitfalls of WB for determining antibody specificity (Holmseth et al. 2006).
7. When the antigen is a peptide, the pure peptide may be able to compete with the antibody for binding antigen. The amount of labeling should be reduced or eliminated when the peptide and antibody are added together to the sample to be labeled.
  Problems: *This pre*-*adsorption approach may block not only ‘specific’ antigen binding to antibody but also the binding of unrelated, cross*-*reactive epitopes* (*‘off*-*target’ antigens*). While this control used to be given much ‘weight’ in terms of evidence of specificity (e.g., Petrusz et al. 1976) more recent authors have illustrated many, often subtle examples of antibodies that are found to be unspecific since they show labeling (and often bands in WB) in KO animal tissues but nevertheless ‘pass the pre-adsorption test’; in this case, the peptide blocks both ‘specific’ and cross-reactive antigens (Holmseth et al. 2005; Burry 2011; Swaab et al. 1977; Saper and Sawchenko 2003; Pradidarcheep et al. 2009). The affinity of the antibody for an antigen in a protein may also be much higher than for the free peptide. In that case, the antibody may be specific but the pre-adsorption test fails to reveal it.
8. Two or more independent antibodies made against X should give the same or similar labeling pattern. If these are peptide antibodies, ideally these should be against non-overlapping sequences in the protein (Rhodes and Trimmer 2006). Problems: *Two or more antibodies may recognize the same antigen, as well as the same, unrelated cross*-*reactive molecule* (Holmseth et al. 2012).
9. The pre-immune serum should not show a reaction. This is important for antigens from infectious organisms that may have infected the immunizing animal before the immunization. Also when host cells infected with these infectious organisms are labeled, part of the signal can be due to cross-reactivity with the pathogen. This is often difficult to notice, especially with light microscopy and when a host antigen is expected to localize close to the pathogen, for example on the phagosome or vacuole membrane surrounding a pathogen. We have observed cross-reactivity in particular with rabbit primary, as well as secondary polyclonal antibodies with infectious agents such as *Mycobacterium* spp., *Rhodococcus* spp., *Plasmodium* spp. and *Babesia* spp. (Repnik and Griffiths, unpublished data).
  Problems: *Except for antibodies against infectious organisms this control only provides crude information that the antibody reactivity is somehow related to the original antigen that was used for immunization.* Pre-immune serum is anyhow not readily available from companies that sell antibodies.
10. At the tissue level, the combination of immunocytochemistry and in situ hybridization can provide important supporting information about the antibody when the localization of mRNA for a protein correlates with the localization of an antibody (Rhodes and Trimmer 2006) but see (Burry 2011). If the cells/tissue of interest can be isolated, the use of RT-PCR can also be valuable to detect the gene product at the mRNA level.
  Problems: *The methods used for* in situ *hybridization/PCR are generally different and harsher than those used for immunolabeling* (Burry 2011). *The presence of mRNA does not guarantee the expression of the protein of interest or indeed the level of expression that might be needed for the detection of the protein of interest. The level of mRNA expression may be very much lower and possibly undetectable.*

Even in the best cases, with optimal specimen preparation, an accessible antigen and a highly specific, high affinity antibody there is still a threshold of antigen below which it cannot be detected (Griffiths et al. 1993; Fritschy 2008). Given the unpredictability in antibody–antigen interactions in situ such as on sections it is difficult to estimate what this lower level of detection can be. If the amount of primary antibody that binds a section is relatively low, a positive signal for this bound antibody can be detected more sensitively using small fluorochrome labeled secondary antibodies and immunofluorescence than they can by those attached to bulkier gold particles; the latter are known to be less sensitive as they increase in size (Schwarz and Humbel 2007). If one is below the detection threshold with the primary antibody for the antigen, the only way out is to over-express the protein. However, that approach brings its own problems. If the antigen is in a defined structure like the nuclear pore complex, where each pore can only accommodate a defined number of proteins, increasing the amount of these proteins may lead to its mis-localization to the cytoplasm, ER or nucleus (Soderqvist et al. 1996; Bastos et al. 1996). Another way is to increase the signal by collecting it from as large an area of sample/section as is possible. This is possible with gold particle labeling in EM.

## The provenance of the antibody and the biology of the system

Before one decides to try to localize an antigen by ICC one must have a rational biological question, or questions to address. One must know something about the antigen even before one attempts to make, or buy an antibody. The more one knows from the outset the easier it is to have *a starting hypothesis* as to where the antigen might be localized, and also important, where it might not be expected. This facilitates the evaluation of the antibody if some structures (not expected to contain the antigen) do not label while other structures (predicted to contain the antigen) do label. In the simplest instance, an antibody against a membrane protein should not label the nuclear matrix. It is obviously much easier to do this at the EM versus LM level because more information is available in the system. The EM approach can also facilitate the selection of the optimal concentration of antibody. Of course, if the starting hypothesis is wrong, the situation becomes more complex and other information must be sought.

The predicted protein sequence might suggest clues. Thus, a membrane protein, or from a sequence-predicted membrane protein, would be expected to be localized on membranes. Similarly, the presence of a secretion signal sequence would predict a localization outside the cytoplasm (e.g., ER, Golgi, endocytic organelle, extracellularly).

These days, perhaps the most common route to an antibody is to make peptides from the known sequence. There are a number of algorithms available based on known experience of what kinds of sequences are most likely to give an immune response, e.g., Expasy (http://www.expasy.org/tools/) IEDB website (http://tools.immuneepitope.org/tools/bcell/iedb_input), or Antigen Profiler (http://www.pierce-antibodies.com/custom-antibodies/peptide-design-antigen-profiler.cfm). In general, sequences predicted to be on the 3-D surface of the protein are favored. Many commercial companies now offer sequence prediction and peptide synthesis as part of their routine service. As has been pointed out by many authors and editors of Journals, it is imperative to describe the sequences used when one publishes about the use of an antibody, as well as all other relevant background information (Fritschy 2008; Saper 2005). If the antigen has been purified or is part of a more complex mixture the details of how it was prepared and used to immunize the animal needs to be specified, as should the question whether the immunocytochemical product used was a monoclonal or polyclonal antibody. If the latter, did one use the serum, IgG fraction or affinity-purified antibody after passing the serum over a column, or using affinity beads bound to the original antigen or, perhaps, protein A? As pointed out by Rhodes and Trimmer (2006) there is no hard and fast rule for determining if purification is needed, but in general the specificity of polyclonal antibodies improves with purification. It must be pointed out that given the harsh reagents needed to remove the bound antibodies from columns, one may fail to elute the highest affinity antibodies that, in contrast, would be present in the antiserum.

When one makes an antibody from a peptide the carrier used to conjugate the peptide is important for immunogenicity but may cause problems with respect to retrieving the most specific antibodies from a serum. The most common method is to affinity purify the serum over a column with the peptide immobilized on beads, a procedure that is most efficient when the peptide is conjugated to a protein, such as keyhole limpet hemocyanin (KLH; Daniel Louvard, personal communication). KLH, a large copper-containing protein of multiple subunits of 350Kd has become popular as a carrier protein because it is highly immunogenic and expressed only in arthropods and molluscs. It is therefore evolutionary far from mammals; this is considered important in reducing the incidence of false positive reactions that are more likely to occur with mammalian carrier proteins such as BSA (bovine) or ovalbumin (Chicken eggs).

## Polyclonal versus monoclonal antibodies

Both polyclonal and monoclonal antibodies are widely used for immunocytochemistry. In general, polyclonal antibodies are more often successful for immunocytochemistry than monoclonals. This is to be expected since polyclonals are derived from a family of B cells and recognize many different epitopes on the same protein, whereas monoclonals, derived from a single B cell clone are specific for single epitopes. In our experience, the best monoclonals are those available as the culture supernatants of the hybridoma cells; these are diluted no more than 1:10 with the blocking solution. A big theoretical advantage of monoclonals is that each batch must have the same specificity and the supply is unlimited. In contrast, no two batches of polyclonal antibody can be expected to be identical and the supply of any one batch is limited. This lack of reproducibility and sustainability of the supply is a severe problem with commercial sources of polyclonal antibodies.

## The problems with commercial antibodies

According to Bordeaux et al. (2010) there are now over 180 commercial companies that sell around 350,000 antibodies to the scientific community (http://www.antibodyresource.com/onlinecomp.html). This is one of around 20 important publications that have started to question the fact that has long been apparent to anyone with experience in purchasing and using commercial antibodies, especially for immunocytochemistry: a significant fraction of commercial antibodies appear to be ‘no better than PBS’ (Couchman 2009). Since most of the critical comments have been in particular fields, such as neurobiology, physiology, pathology and cancer diagnostics we suspect most of these publications will not have been seen by many cell biologists, even those that are heavy users of commercial antibodies. The facts are collectively very disturbing—at worst almost entire fields of research have been led down wrong paths by use of a few prominent commercial antibodies that were later discovered to be nonspecific or of dubious specificity. One important thread that links all of these, mostly recent publications is the powerful use of knockout animals which, despite the theoretical problems raised above, are increasingly being accepted as the ‘gold standard’ for antibody specificity. Other criteria summarized above also play important supporting roles in this ongoing ‘detective story’.

One issue of Naunyn-Schmiededberg’s Archives of Pharmacology has six articles that collectively deal with, mostly widely used commercial antibodies against an array of membrane receptors that are demonstrated to lack the required level of specificity for immunocytochemistry [summarized by Kirkpatrick (2009)]. The first paper (Jositsch et al. 2009) focused on five types of muscarinic receptors (M1R-M5R) that bind acetylcholine. They tested 24 commercial (four different companies) and ‘home-made’ antibodies using IF on cryostat sections of wild type mice and mice deficient for the five receptor sub-types. Sixteen of these antibodies were tested in detail using a total of 456 different antibody/different protocol combinations. Together with four different dilutions of each antibody this meant they had to test over 1,800 different conditions. Kirkpatrick states ‘With two exceptions (anti-M2R), however, all antibodies produced identical immunohistochemical labeling patterns in tissues taken from corresponding gene-deficient mice, even when the pre-absorption control (with peptides) suggested specificity’ (the latter problem has been discussed above-criterion 8).

In the same Journal issue, Lu and Bartfai (2009) focused on immunolocalization of G-protein-coupled receptors for the neuropeptide galanin (GalR1 and 2) and took advantage of KO mice and commercial polyconal antibodies against peptides specific to the two receptors from three different companies. Strikingly, all the antibodies gave essentially indistinguishable pattern of labeling on cryostat sections and Western blots between WT and KO mice. A similar result was seen in the next article by Everaerts et al. (2009) who tested three antibodies against the transient receptor potential cation channel vanilloid sub-family member (TRPV-1). All antibodies from three different companies gave a similar labeling pattern in sections of KO and WT mice.

Three more papers in the same issue provided Western blotting data revealing that antibodies to muscarinic and adrenergic receptors (Jensen et al. 2009) gave multiple bands by Western blotting in KO tissue. An over-expression, transfection approach was used for antibodies against dopamine receptors by Bodei et al. (2009) who came to the same conclusion: lack of specificity. While it must be emphasized again that lack of specificity on blots is not the same as proof of lack of specificity for immunolabeling, the fact remains that the majority of commercial antibodies are claimed above all else to be specific by Western blotting. It must also be pointed out that in the four studies combined, a total of over 30 antibodies were deemed to lack specificity.

In a more recent, comprehensive analysis of six commercial antibodies against the angiotensin II AT_1_ receptor from three companies Benicky et al. (2012) compared WT and KO mice. By the criteria of tissue section IF, by expressing (or not) antigen in cells lacking antigen and by Western blotting all six antibodies were concluded to be nonspecific. Again, these authors found that pre-absorption of antibodies with the immunizing peptides led to a loss of labeling (false negative data) that was not consistent with the (more convincing) other results. ‘A related problem, not only in datasheets of scientific publications is the ‘trick’ of illustrating only a cut-out band of interest and not showing the annoying ‘additional bands’. Or as stated by Pradidarcheep et al. (2009) in their study of muscarinergic and adrenergic receptor localization ‘our immunoblots (based on whole cell extracts) show numerous bands for each antiserum, whereas those shown in a supplier’s catalog often show a single band only’.

The P2X7 receptor that binds extracellular ATP on cell surfaces has been widely analyzed using antibodies against the intracellular and extracellular domains of the mouse receptor from the company Alomone laboratories. However, a detailed review by Anderson and Nedergaard (2006) summarizes the body of data showing that these antibodies gave a selective labeling and the presence of multiple bands in tissues on KO animals.

Yu and Hill (2013) addressed the specificity of related receptors to UDP, P2Y6 receptor in bladder smooth muscle. ‘Three commercially available antibodies to P2Y6 receptor gave clean bands on Western blot which were eliminated by specific peptide competition. Two of the three also immune-stained bladder smooth muscle cells while leaving adjacent interstitial cells of Cajal unstained. However, attempts to validate the specificity of these antibodies by performing the same assays on bladders from P2Y6 knockout mice were unsuccessful. In Western blots, all three antibodies bound similar proteins in both wild type and P2Y6 knockout tissue. Immunostaining of knockout tissue sections also showed no difference in staining patterns or intensity’. This problem with commercial antibodies is summarized also in a recent article by Buckingham (2014; http://www.labtimes-archiv.de/epaper/LT_14_01/files/assets/common/downloads/LabTimes_2014_01.pdf).

Data sheets provided by commercial companies often lack important information and can even be misleading. Specificity for antibodies is definitely related to the concentration of IgG used experimentally (1–10 μg per ml is a reasonable range). Hence a preliminary set of experiments is needed to test and adjust cautiously the optimal dilutions. To demonstrate antibody specificity, companies often use cells overexpressing the protein; the abundant protein gives a single band on the blot, whereas detection of endogenous proteins may be a lot more difficult and the problem of poor specificity of the antibody becomes more prominent. Data sheets can also show results obtained several years ago. In particular, in the case of polyclonal Ab, it is not likely that the same serum is still available, so the image in the datasheet may be unrepresentative of the reagent actually available. Fluorescent images of immunocytochemistry results are shown at low magnification, so the signal appears strong and the whole cell is glowing. But these images fail to demonstrate subcellular localization and thus give no information about the specificity of the signal.

Can one do anything to avoid buying useless antibodies? The review by Bordeaux et al. (2010) is an eye-opener into this, obviously very annoying issue. In an in silico exercise, they decided to select seven companies selling antibodies against the randomly selected molecule AMP kinase-β 1, a cytoplasmic enzyme involved in energy metabolism, simply in order to compare the level of background information available. Even though the commercial sources were not identified the difference between the worst (company 1) and the best companies (6 and 7) was especially revealing. Company 1 in fact was more prominent for what it did *not* reveal, such as the sequence of the immunizing peptide [this is apparently not an uncommon problem—see Saper (2005)] In contrast, companies 6 and 7 provided a wealth of important information in their accompanying data sheets, including publications using the antibodies, examples of use of KO cells, Western blotting and immunocytochemistry under a variety of conditions and giving recommended conditions such as antibody dilutions for different approaches. This makes the important point that it pays to do one’s homework before deciding where to buy an antibody! This point is also emphasized further by Kalyuzhny (2009) who writes an interesting article on the issue of commercial antibodies from the perspective of the manufacturer. He advises the purchaser to even contact the companies for advice and for knowledge of available technical support before buying the antibody. It may even be possible to strike a deal with the company in which one pays only after initial testing of a small aliquot or one receives rolling credit if antibodies do not work in exchange for details of the methods used. Of course, given the theoretical background we have discussed and the almost complete unreliability of a priori prediction of how an antibody will behave, there is no way that even the most reliable and professional manufacturers can provide a complete guarantee of success, any more than one can do so when one prepares one’s own antibodies.

## EM: an effective quantitative tool for use with commercial antibodies

Given the challenging state of the art for commercial antibodies we have described above, it is incumbent on the investigator to make a smart choice and careful use of these tools. In the context of the foregoing discussion, we will now outline how best to evaluate, test and use antibodies in immuno-EM, a method that is currently the technique of choice for questions related to protein localization.

Although immunofluorescence microscopy (IF) of cells and tissue sections is perhaps the dominant approach used in most cell biology labs today, there are a number of advantages in using immuno-EM on thin sections. First, it is generally accepted that one sees more ‘details’ by immunogold labeling when compared to the IF (or peroxidase-based) methods used in light microscopy. Second, when monitoring immunogold labeling on cell or tissue sections, one sees the immunogold signal *and* also the profiles of all the structures in the cells (the reference space). By contrast, with IF one analyses the localization of one or two antigens (the IF signal) against a ‘black’ background, The increased structural information provided by EM then makes it easier to decide where *all* the gold particles are localized; for example an antibody against a membrane protein should be localized within two 10 nm particle widths from a membrane (Griffiths et al. 1993). An additional advantage is the digital nature of the gold signal which makes it easy to count and quantify, whereas quantitative analysis of light microscopy immunocytochemistry is problematic. The combined use of digital, all or none, immunogold labeling, combined with the high precision in allocating them to defined structures make this system ideal for minimally biased and efficient quantitative analyses using stereology-based methods (Lucocq 2008, 2012).

It should be noted that, even for IF, the use of thin section analyses of plastic or thawed cryo sections can provide more information than do standard (relatively thick) confocal slices. This is because one can use phase contrast and other methods to analyze the reference space (Schwarz and Humbel 2007). Furthermore these extremely thin sections will also provide good vertical and horizontal resolution, and nonspecific background labeling is easier to notice than in whole mount IF. An additional advantage is that fluorochrome-conjugated reagents are smaller than colloidal gold reagents and so the labeling is less likely to fail because of steric hindrance, often a problem with immunogold labeling (Schwarz and Humbel 2007). In our experience, on-section IF is also very suitable to test antibodies because the cell interior is uniformly exposed by sectioning. In our view, IF on sections is an under-used yet important ancillary method for initial assessment of antibodies.

We will now briefly summarize relevant aspects in evaluating and testing new antibodies for use in immuno-EM. We will then go on to describe methods we have developed for assessing and quantifying patterns of immunogold labeling and finally describe a method for assessing specificity.

## Evaluation of commercial antibodies

**Fig. 2:**
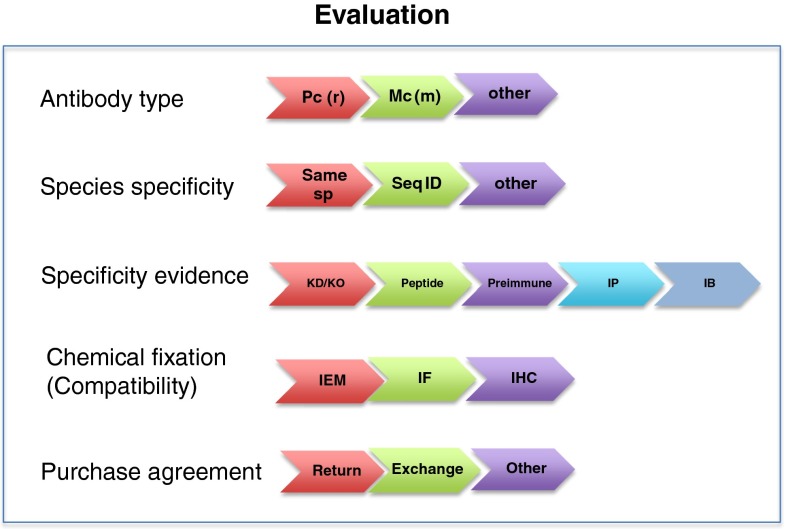
Evaluation of commercial antibodies. The figure summarizes factors to consider prior to purchasing antibodies with the most important characteristics listed to the left of each row. For the purposes of immuno-EM, polyconal (Pc) antibodies are preferred because they bind multiple epitopes and have maximum likelihood to withstand aldehyde cross-linking used for specimen preparation. Rabbit (r) antibodies are preferred for immuno-EM because they interact with the general second-step reagent protein A-gold. Monoclonal antibodies (Mc) from mouse (m) can be localized with well-characterized intermediate antibodies or antibody gold complexes. Species specificity is often stated by companies but likely interactions of antibodies from other species can sometimes be assessed before purchase by comparing published gene/protein sequences of intended target epitopes with company-specificity data (Seq ID). The main issue considered here is antigen specificity and a powerful determinant is data showing that labeling is reduced or ablated using knockdown/knockout (KD/KO). This control is rarely quoted. Some supporting evidence may come from peptide inhibition experiments or more frequently by light microscopy pictures showing the pattern of labeling (IF/IEM). Specific binding to the target in independent biochemical assays (immunoprecipitation, IP; immunoblotting, IB) are less valuable for using antibodies for immune-labeling. Further factors to consider before purchase are the preservation of antibody–antigen interactions after fixation/processing methods such as immuno-EM (IEM) immunofluorescence (IF) or paraffin immunocytochemistry (IHC). Finally, some antibody companies are beginning to offer purchase agreements, which include refunds or exchange deals for antibodies that do not produce results, or deals offering antibodies or reduction in costs in return for data obtained using the purchased antibody

When searching for an antibody from a commercial source one is faced with the dilemma of which characteristics to consider as most important (summarized in Fig. 2). As already discussed, polyclonal antibodies are to be preferred over monoclonals because the multiple epitopes are more likely to survive the processing procedures of immuno-EM. Furthermore, many polyclonal antibodies are made in rabbits, and bind protein A-gold, which is a robust and dependable second-step reagent. A second factor in antibody choice is species specificity for which commercial information can be based on wide variety of evidence. At one end of the spectrum, a company may simply state the species from which the antigen (or its sequence or peptide) used to generate the antibody is derived; at the other the company may additionally provide biochemical or other evidence that the antibody actually interacts with the antigen from that species. Whatever the evidence present the species information can nevertheless be very useful for the customer who can investigate compatibility in other species independently by screening databases for sequence similarity to original antigen.

As we have emphasized here, the key characteristic of antibody specificity is usually poorly evidenced for many commercial antibodies. Evidence for specificity is mostly provided by immunoblot (or immunoprecipitation) data, although this there is an increasing trend for this to be supported by pictorial ‘pretty-picture’ evidence for a staining pattern by immunofluorescence or immunocytochemistry on paraffin sections. Indeed, when the distribution resembles the ‘predicted’ organelle distribution from experimental or sequence data this is encouraging, although as we have already discussed, not necessarily conclusive. To date, it is extremely rare for patterns of immuno-EM studies to be reported, but independent searches in the literature can be very useful to identify studies in which the same antibodies have already been used. Worryingly, it is also extremely unusual for companies to report results of peptide inhibition studies and sadly even rarer for them to report KD/KO data.

## Quantifying gold labeling in immuno-EM

**Fig. 3:**
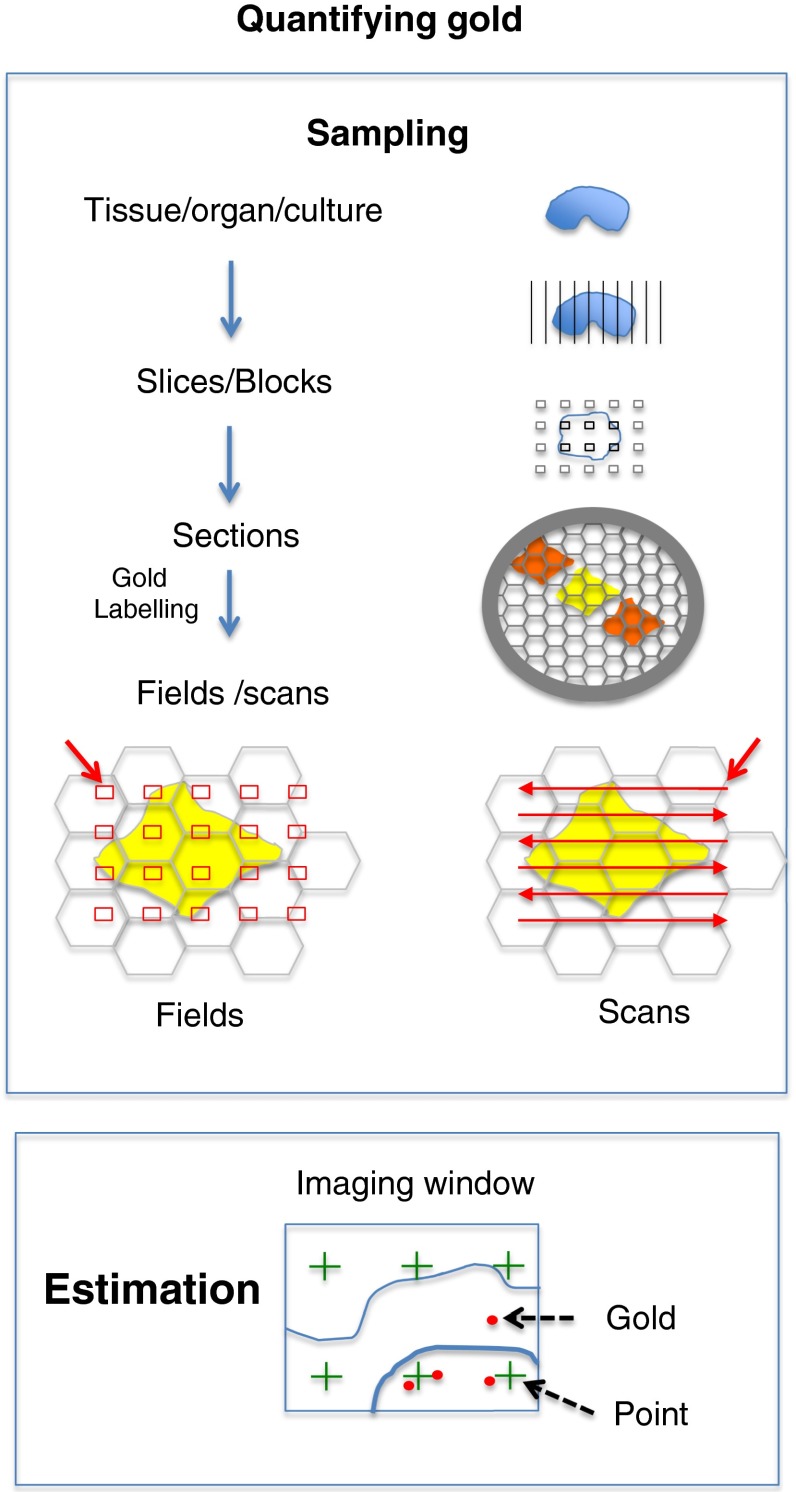
Quantifying gold labeling. In immuno-EM the experiments are done on animals, organs or cell cultures and the estimates of gold labeling should reflect the ‘ground truth’ values in these members of the population, with minimal bias and a high degree of precision. The link between what is actually measured on the EM section and the ‘population’ is made using appropriate sampling schemes. The basic sampling approach is called simple uniform random but a powerful modification to simple random sampling that is easy to implement is systematic uniform random sampling (SURS; Lucocq 2008, 2012). As illustrated here the systematic samples (slices, blocks, sections or microscopic fields) are spread through the objects of interest but always with a random start to ensure unbiasedness (see Lucocq 2008). For example the microscopic fields (*bottom left*) or scans (*bottom right*) are placed in a systematic array across the sample (10–20 in number) starting from a random location (*red arrows*). Estimations may include distributions of gold counts over cell structures or the density of gold counts. Gold counts can be made most rapidly by scanning and viewing directly at the electron microscope or on a viewing screen via a CCD camera whereas density is more conveniently carried out on microscopic fields. Density can be expressed as gold particles per unit area of structure profile or gold particles per unit length of membrane trace. In the example illustrated here the area is estimated by applying a systematic point array placed in a systematic random fashion. The sum of the number of points ‘landing’ on features of interest multiplied by the area associated with each point in the lattice is an estimate of the total profile area. The density of gold labeling can be then estimated from the number of gold/area of the feature [see Lucocq (2008) for more details and for the method for estimation of membrane profile length]. Rather modest numbers of point hits and gold particles are needed (100–200 of points or gold particles in total per experimental condition or animal)

The method of choice in immuno-EM is on-section labeling because it ‘opens up’ cell compartments by preparing an ultrathin section from a biological specimen. Within the section compartments and structures containing the antigens of interest are then exposed to the labeling reagents that are localized by the (quantifiable) gold label. If adequate contrasting with heavy metals is used, a wide range of compartments/structures can be displayed to which the gold particles can then be assigned. In this process of quantitative immuno-EM there are two important considerations to consider. The first is how to sample correctly and the second is how to obtain quantitative readouts of the gold labeling once the section has been labeled. These two aspects are illustrated in Fig. 3.

While the gold signal is quantified by EM using micrographs or scans, every effort should be made to make the final estimates representative of the intact specimen. The goal is to ensure that the quantities obtained from the gold signal or structures will faithfully report on the ‘ground truth’ values found inside of the organism or experimental cell culture. Each micrograph usually represents a very tiny portion of a section, which is in turn derived from blocks, fragments or slices of the organ or cell pellets. The solution is to use rigorously applied random sampling protocols throughout this multistage process. Simple random sampling ensures that all parts of the specimen have an equal chance of being included in the section, thereby giving all possible gold particles and parts of each structure an equal chance of being selected for quantification. However, there is an important modification to random sampling, which can improve the efficiency of the estimates especially when it is applied to heterogeneous biological samples used in cell biology. This modification is called *systematic uniform random sampling* or SURS (Lucocq 2012). SURS selects an array of samples that is spread evenly through the specimen/section with random placement of the array, ensuring unbiasedness of the procedure. In cellular systems, the use of SURS often yields estimates that are more precise than those obtained using simple random sampling and this method of sampling is also easier to carry out. SURS can be used for sampling, organs, tissues, cells and organelles as well as for collection of data on the section itself [Fig. 3; see also (Lucocq 2008) for more detailed discussion].

**Fig. 4:**
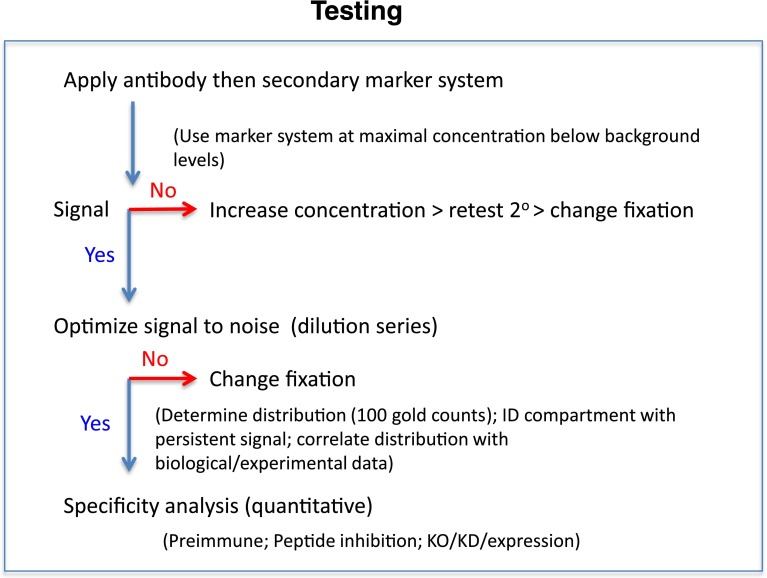
Testing antibodies for immuno-EM. Purchased antibodies for use in immuno-EM should be applied in the chosen labeling system in combinations with appropriate blocking agents. In addition a well-established secondary marker should be used at a concentration that is the highest that is compatible with acceptably low background staining. If no signal is obtained, then the antibody concentration should be increased or the fixation modified to replace glutaraldehyde (more extensive cross-linking) with formaldehyde. Once a signal has been observed then a dilution series allows signal to noise to be assessed. The simplest method for assessing the distributions across a number of compartments is by counting a total of 100–200 gold particles over 10–20 micrographs (Lucocq et al. 2004); and patterns of preferential labeling across the dilutions series can be evaluated using Chi square analysis. The distribution analysis often reveals compartments in which proportions of total gold label they hold increase as the primary antibody becomes more dilute (Lucocq and Hacker, unpublished observations). These compartments are likely to hold specific labeling. By comparison distribution analysis may reveal compartments over which the proportion of total labeling falls with dilution of the antibody. These compartments are likely to display ‘nonspecific’ labeling. As candidate labeled compartments emerge then the biological/molecular information about the antigen can be compared and if necessary the labeling, once optimized in this way, can then be analyzed formally for specificity as detailed in Fig. 5

Once the section has been generated and labeled with gold reagents, the simplest way to express immunogold data is to assess the distribution of labeling over a range of organelles/compartments using a SURS-based approach. The principle is to count and assign each gold particle to an identifiable compartment (if necessary, a class referred to as ‘non-identified’ structures may also be included). In one approach the ‘counts’ are carried out by viewing the labeled sections directly on the electron microscope viewing screen or via a camera display. The counts are made in series of scans, which are made across the labeled section with a random start (SURS; Fig. 3). Alternatively images can be recorded in a series of imaging fields or micrographs (Fig. 3). In this ‘micrograph’ method, images are recorded at between 10 and 20 systematically placed locations across a single gold-labeled section, again with a random start (SURS). In either approach, the magnification is set at a minimum value that allows clear identification of both the gold particles and the structures of interest. Previous work has revealed that when 10 or so compartments/structures are considered, a total of 100–200 gold particles are required to produce a consistent distribution pattern (Lucocq et al. 2004). If more precision is required for individual compartments, then more particles may need to be quantified. If one stringently follows the unbiased counting rules and has a consistent labeling protocol, it is striking how quantitatively reproducible the results are from one EM grid to the next, and from experiment to experiment by this simple approach. Such an approach conveniently provides simple and rapid way to assess any preferential distribution of labeling through the initial evaluation process (as suggested and outlined Fig. 4).

Finally a second type of readout is gold labeling intensity which can be assessed by combining gold counts with stereological estimations of structure profile parameters, such as length or area (Lucocq 2008). The gold density provides an important tool in assessing specificity (see next section).

## Assessing specificity of gold labeling

To address the question of specificity, a method was recently developed, which utilizes cells in which an antigen can be knocked down (KD) or knocked out (KO; Lucocq and Gawden-Bone 2010; Hacker and Lucocq 2014). This gives an opportunity for those elements of labeling that are specifically related to the antigen of interest to be assessed.

**Fig. 5:**
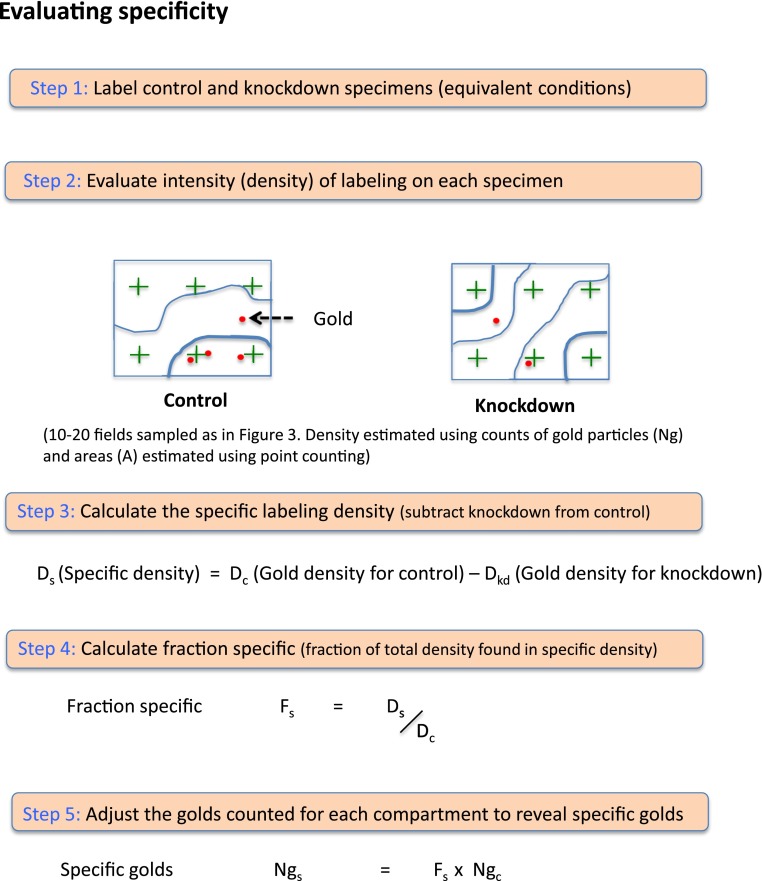
Evaluating specificity in immuno-EM. The principle is to compare the gold labeling density over a range of organelles or cell compartments before and after knockdown or knockout of protein expression. Since this comparison cannot be done sequentially on the same specimen (fixation and cell death occur during processing!), it must be done on two separate specimens which are then compared (Step 1). For a compartment or structure containing ‘specific labeling’ the gold labeling density will be higher over the control specimen (full expression) than over the same compartment after knockdown (contains less of the target protein). It is possible to subtract the labeling density obtained from the KD or KO specimen from the density obtained from the control (Step 2). This provides the investigator with an estimate of labeling density that can be attributed to the antigen (Step 3). Notice that for a knockdown (rather than a knockout) this ‘specific density’ will be a minimal estimate because the suppression of expression will generally be incomplete. SURS is especially important here because cell to cell variation in the population can occur and this must be ‘sensed’ or accounted for in the result. It is important that the processing of each specimen (control and KD/KO) must be as similar as possible, and rigorous sampling must be applied to remove error. We recommend generating specific labeling values from 3 to 5 individual pairs of control and knockdown samples. Once the specific density is known the fraction of the control labeling over a compartment that is specific can be found (Step 4). This ‘fraction-specific’ expresses the likelihood that an observed gold particle is specific. Finally using this fraction-specific the original distribution of gold particles (which contains nonspecific and specific labeling) can now be adjusted to express the distribution of specific gold particles (Step 5). The distribution of specific labeling is found be multiplying the fraction-specific with the original (control) counts. [see refs Lucocq and Gawden-Bone (2010) and Hacker and Lucocq (2014) for more details]

The principle (see Fig. 5) uses two specimens that are fixed, processed, sectioned and labeled for the antigen of interest under identical conditions. One specimen is left untreated, and the other contains cells or tissues in which expression of the antigen has been KD or KO. In a first step, the density of gold labeling over a range of compartments is estimated using stereological techniques under both conditions. The density is expressed as the number of gold particles per membrane profile length or organelle profile area (Griffiths et al. 1993). The next step is to calculate the difference between the density values for control and the KD/KO for each individual organelle type. This generates a measure called the ‘specific density’ which assesses the portion of the control gold labeling density that can be attributed to the presence of the antigen (notice that when the expression of the antigen is reduced rather than ablated the specific density can be an underestimate of the full extent of specific labeling for a particular compartment). From these data, it is then possible to assess the proportion of the labeling over each compartment that is attributable to the antigen and therefore the probability that a gold particle might represent the antigen (also called the fraction-specific). As an example if the fraction-specific is 0.8, then there is an 80 % probability that an observed gold particle located of a compartment is specific for the antigen of interest. Once this fraction-specific is known, it is straightforward to correct the initial labeling distribution to yield a specific labeling distribution profile. This is a simple matter of multiplying the fraction-specific with the raw (initial) gold signal for each compartment. Again if knockdowns are incomplete then the estimate of specific labeling will be an underestimate. It is worth noting that the above scheme can also be applied for assessing specific labeling that is generated by expression of tagged proteins (see Lucocq and Gawden-Bone 2010; Hacker and Lucocq 2014).

## Conclusions

In summary, it must be accepted that trying to ‘prove’ that an antibody is specific for a given antigen is a complex, multi-faceted issue, associated with many pitfalls. Given that an increasing number of antibodies are purchased from companies extreme care needs to be exercised when selecting and testing antibodies from commercial sources; however, the risk of obtaining ‘duds’ may be reduced by paying attention to a few key pointers that we have discussed. While the long road between antigen and antibody is a rocky one, more judicious use of specificity controls by commercial companies would be highly desirable for improving the reliability and usefulness of these powerful tools for cell biology and related disciplines. At least for EM some effective methods are now available for establishing quantitative estimates of labeling distributions, and for determining more accurately how much of the labeling is due to the interactions of the antibody with the intended target. Given the commercial importance of the market and the extent to which researchers are dependent on their products, it is now high time for the companies to put more effort in trying to ensure that more of the estimated 350,000 antibodies on the market do what they are claimed to do.
